# Supplementary material for: Wnt Signaling Is a Major Determinant of Neuroblastoma Cell Lineages
Source: Front Mol Neurosci. 2019 Apr 16;12:90. doi: 10.3389/fnmol.2019.00090 (PMC6476918; doi:10.3389/fnmol.2019.00090)
Supplement: FIGURE S1 — (A) Correlation between MES signature and WMG-1 and (B) Kaplan-Meier survival analysis of MES genes in Kocak(649) data set (GSE45547). [file Presentation_1.pdf]

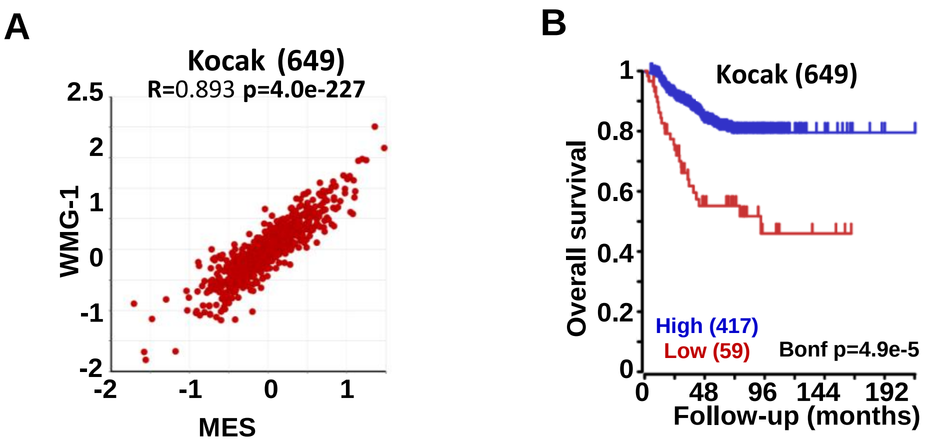

Supplementary figure 1.

(A) Correlation between MES signature and WMG-1 and **(B)** Kaplan-Meier survival analysis of MES genes in Kocak(649) data set (GSE45547).

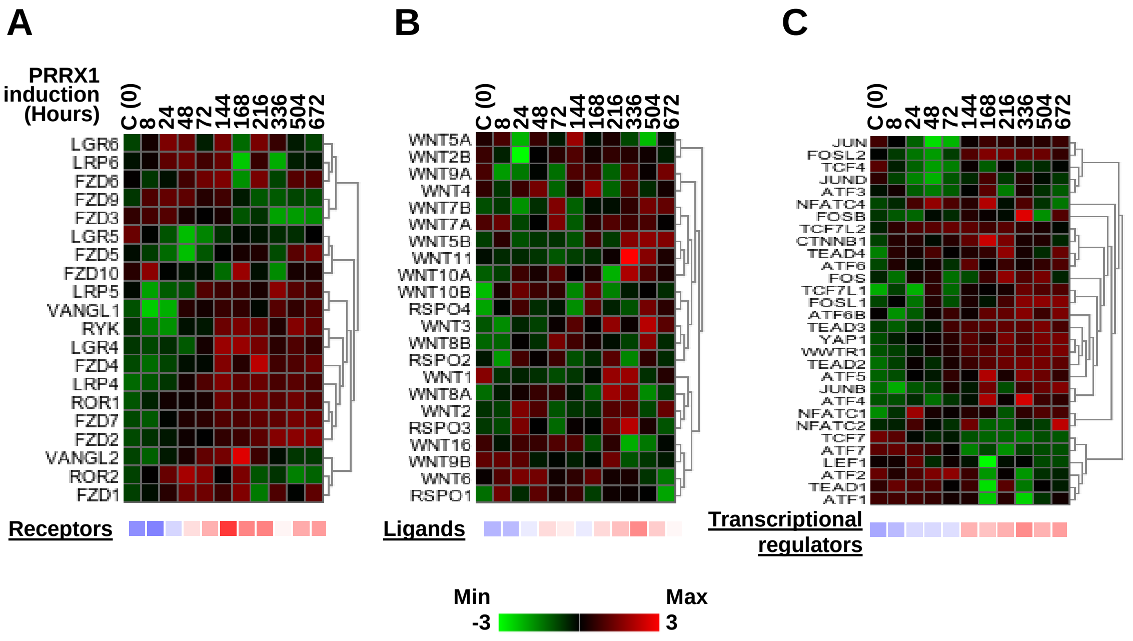

Supplementary figure 2.

Heatmaps showing the regulation of the Wnt receptors (A), ligands (B) and transcriptional regulators (C) in PRRX1-induced SK-N-BE(2)-C cells (GSE90804). In addition to KEGG-derived Wnt pathway members, we included other known regulators as well. *LGR4-6* were added to the receptor group and *Rspo-1-4* to the ligands **[14]**. Wnt-associated transcriptional regulators also included *YAP1*, *WWTR1*, *TEAD1-4*, *ATF1-7*, *FOS*, *FOSB*, *FOSL1/2*, *JUNB*, *JUND* and *TCF4* **[9, 11]**.

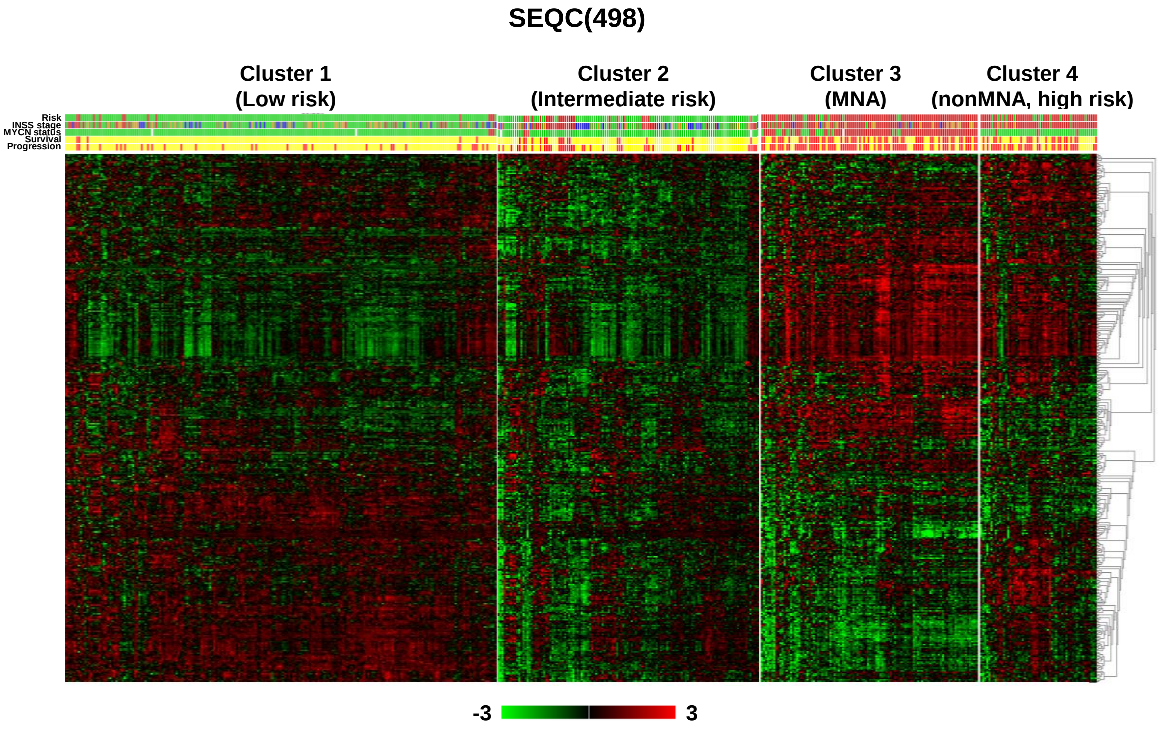

Supplementary figure 3.

Expression of ADRN signature genes in prognostic, Wnt-related NB clusters in SEQC data set (GSE62564) revealed two subgroups that correlated with risk status.
