# Supplementary material for: Wnt Signaling Is a Major Determinant of Neuroblastoma Cell Lineages
Source: Front Mol Neurosci. 2019 Apr 16;12:90. doi: 10.3389/fnmol.2019.00090 (PMC6476918; doi:10.3389/fnmol.2019.00090)
Supplement: TABLE S1 — Oligonucleotide primer sequences for qPCR. [file Presentation_2.pdf]

Gene	Primer	Sequence
EYA1	EYA1-F	ACTTACCAGCTTCAAGAACCG
	EYA1-R	ATTTCCCATCTGAACCTCGAC
PHOX2A	PHOX2A-F	ACTCGTGCGTGGCGG
	PHOX2A-R	CTGGGAAGAACTTGTAGGGCA
PHOX2B	PHOX2B-F	AAACTCTTCACGGACCACGG
	PHOX2B-R	CTCCTGCTTGCGAAACTTGG
PRRX1 isoform b	PRRX1b-F	CATCGTACCTCGTCCTGCTC
	PRRX1b-R	CAGGGCTATTGTTGGCACAT
TFAP2B	TFAP2B-F	TGAAGATGCCAATAACAGCGGCA
	TFAP2B-R	GGAGCAAAACACCTCGCCGGT
WWTR1	WWTR1-F	GTCCTACGACGTGACCGAC
	WWTR1-R	CACGAGATTTGGCTGGGATAC
